# First Study to Describe the Prevalence of Porcine Reproductive and Respiratory Syndrome Virus and Porcine Circovirus Type 2 among the Farmed Pig Population in the Hong Kong Special Administrative Region

**DOI:** 10.3390/vetsci9020080

**Published:** 2022-02-14

**Authors:** Kate J. Flay, Dan A. Yang, Sze Chun Choi, Joyce Ip, Song H. Lee, Dirk U. Pfeiffer

**Affiliations:** 1 Department of Veterinary Clinical Sciences, City University of Hong Kong, Kowloon 999077, Hong Kong SAR, China; 2 Centre for Applied One Health Research and Policy Advice, City University of Hong Kong, Kowloon 999077, Hong Kong SAR, China; d.a.yang@njau.edu.cn (D.A.Y.); lokchoi1991@yahoo.com.hk (S.C.C.); joyceip@cityu.edu.hk (J.I.); songhlee1988@gmail.com (S.H.L.); dirk.pfeiffer@cityu.edu.hk (D.U.P.); 3 College of Veterinary Medicine, Nanjing Agricultural University, Nanjing 210095, China; 4 Department of Infectious Diseases and Public Health, City University of Hong Kong, Kowloon 999077, Hong Kong SAR, China

**Keywords:** porcine circovirus type 2 (PCV2), porcine reproductive and respiratory syndrome virus (PRRSV), prevalence, complex survey, design-based approach, real-time PCR

## Abstract

Infection of pig farms with porcine reproductive and respiratory syndrome virus (PRRSV) and porcine circovirus type 2 (PCV2) causes substantial economic losses globally. However, little epidemiological data of PRRSV and PCV2 in the Hong Kong Special Administrative Region (HKSAR) were available. This pilot study aimed to provide baseline information of the prevalences of PPRSV and PCV2 in the HKSAR. A complex survey was conducted from 3 February 2020 to 11 March 2021 on 29 of the 40 pig farms in the HKSAR, with five pigs each from seven age groups (representing key production stages) tested using a real-time PCR. Evidence of presence of PRRSV European strain (PRRSV-1), PRRSV North American strain (PRRSV-2) and PCV2 was confirmed on 48%, 86% and 79% of farms, with overall prevalences of 7.6% (95% CI: 4.8–10.3%), 12.2% (95% CI: 9.6–14.7%) and 20.3% (95% CI: 14.3–26.2%) in the HKSAR pig population based on pooling results from all pigs across all farms. PRRSV-1 and PRRSV-2 were more prevalent in younger pigs, with the highest prevalences of 32.1% (95% CI: 20.8–45.0%) and 51.5% (95% CI: 38.9–64.0%) for 8-week-old pigs. In contrast, the distribution of PCV2 prevalence across age groups appeared to be more symmetrical, with higher prevalences reported in pigs from 12 weeks old to 24 weeks old but lower prevalences in younger pigs and sows. The results of this study demonstrate that PRRSV-1, PRRSV-2 and PCV2 are widely spread across pig farms in the HKSAR, which indicates that the current farm management and control protocols should be improved. We recommend the implementation of on-farm intervention strategies combined with ongoing surveillance to reduce these viruses, and their consequences, in the HKSAR pig population.

## 1. Introduction

In pig farming systems, respiratory and reproductive diseases cause productivity and consequential economic losses [1,2,3,4]. Respiratory disease is of particular importance in intensive pig production systems [1,5], especially those with natural ventilation systems [6], such as farms in the Hong Kong Special Administrative Region (HKSAR). Globally, pathogen and disease monitoring are utilized in pig farms and slaughterhouses [1,5,7,8], assisting farmers, industry professionals and governments with their decision making [9,10]. Numerous monitoring methods are available, including use of serological and molecular methods, clinical inspections and slaughterhouse surveillance [6,7,9,11]. These methods can be applied both within individual farms and at the wider population level to estimate prevalence of pathogens of interest.

Viral pathogens such as porcine reproductive and respiratory syndrome virus (PRRSV) and porcine circovirus type 2 (PCV2) cause substantial losses to pig producers globally [5,12,13]. PRRSV and PCV2 both cause respiratory disease and reproductive losses, while PCV2 also causes PCV2 systemic disease [14]. The potential economic consequences of these pathogens are substantial and documented in a number of pig-producing countries, including Germany [2,15], the United States [12,16], the United Kingdom [17] and Vietnam [18]. Estimation of prevalence of these viral pathogens typically relies on the use of either serological tests to detect antibodies (e.g., enzyme-linked immunosorbent assay, ELISA) or molecular methods that detect the virus (e.g., polymerase chain reaction, PCR) [11,14]. It is also possible to assess the genetic characteristics of these viruses, commonly used to assess changes in the pathogen, relatedness and spread [19,20,21,22].

The prevalence of PRRSV and PCV2 has been estimated in a number of pig-producing countries worldwide. In Cambodian farms, a PRRSV farm prevalence of ≥85% was reported [19]; 67% of farms were reported as affected via testing of stored serum samples from pig farms in Illinois (USA) [23]; and in Korea, 69% of farms were positive for PRRSV [24]. With respect to PRRSV in different ages of pigs, prevalence is reported to decrease as pigs age [11]. In China, a recent review and meta-analysis used data from 53 studies with an overall of 29,051 samples to estimate the prevalence of PCV2 [25]. The overall prevalence was 46%, while nursery pigs had the highest prevalence and adult sows the lowest [25]. Similar results were reported in Yunnan Province (China), with 61% of samples PCV2 positive and the greatest positive proportions observed in nursery and fattening pigs [20]. In Korea, PCV2 prevalence was reported to increase with increasing age; however, the pigs ranged from 3–26 weeks old (i.e., the oldest pigs are comparable to fattening pigs in the other studies) [26]. The overall farm-positive prevalence was similar to that reported in China, with 52% of farms positive for PCV2 [26].

The present study was undertaken to describe the prevalence of PRRSV and PCV2 in farmed pigs in the HKSAR in order to prioritize pathogen specific research and/or interventions specific to the HKSAR situation.

## 2. Materials and Methods

### 2.1. The Study Population and Pig Production System

In 2021, there were 40 active pig farms (of a total of 43 licensed pig farms) in the HKSAR, with a maximum combined capacity of 74,640 pigs [27]. These farms produce an estimated 115,000 pigs for local slaughter each year [28]. Local production accounts for around 15% of live pigs consumed in the territory, with the remaining live pigs being imported on a daily basis from Mainland China [28]. Until recently, local producers had limited or no access to formal advice on pig health and production from professional sources such as veterinarians and swine consultants. In addition, there were scant data available regarding the health and productivity of farmed pigs in the HKSAR, making it challenging to prioritize pathogen-specific interventions. There had been one previous study in the HKSAR, which reported genomic sequences in 16 PRRSV isolates [29]; thus, it was known that PRRSV was present on at least some local farms. However, there were no data available regarding the prevalence of PRRSV or PCV2, despite their international importance in pig producing systems.

Currently, in the HKSAR, all pig producers must obtain and maintain a Livestock Keeping License (LKL) to farm pigs [27]. Under CAP. 139L, the LKL will only be issued or renewed if the farm has fulfilled all of the license terms, conditions and specified requirements related to livestock keeping, public health and environmental protection, including complying with the Waste Disposal Regulations (CAP. 345A) [30]. Therefore, there is reliable information available regarding the number of farms and total number of farmed pigs in the region.

**Figure 1:**
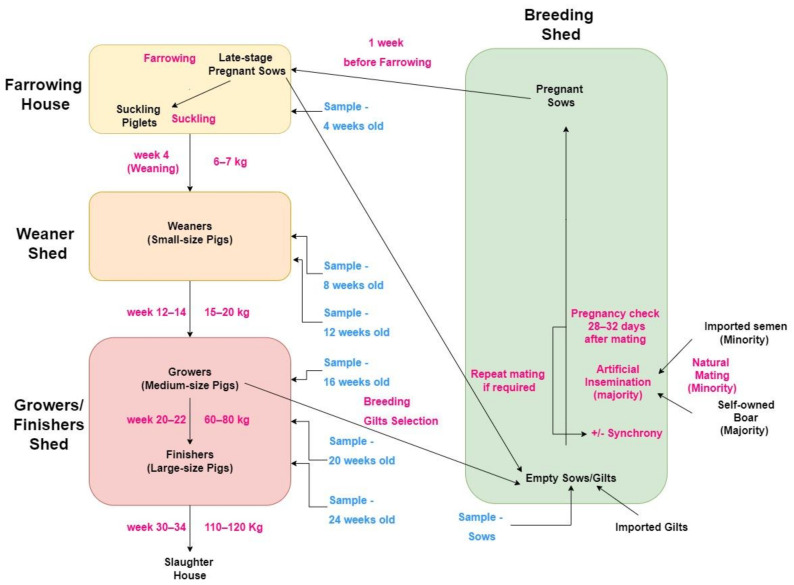
Pig production system in the Hong Kong Special Administration Region showing different production stages and ages and the different age groups that were selected for sampling (‘sample’) in the present study.

In the HKSAR, the average farm size is 212 sows (range 40–500). The typical pig producing system is farrow-to-finish, with piglets born on-farm and reared until slaughter at approximately 120 kg liveweight when pigs are 30–34 weeks old. Pigs are continuously produced, rather than batch production or all-in/all-out systems. Currently, there is no standard feeding protocol for pig farms in the HKSAR, with farmers selecting feeds based on their own farm needs and access to feedstuffs. There are no data available about specific on-farm feeding protocols for each farm. A number of farms (approximately 70%) import live gilts from Taiwan twice yearly to replenish their breeding stock, with the F1 crossbred between Landrace and Large White breeds. The pigs are slaughtered in government-managed slaughterhouses, one in Sheung Shui and the other in Tseun Wan [31]. The pig production system in the HKSAR is summarized in Figure 1.

In the HKSAR, limited vaccine records are kept on most farms, and as such, the individual vaccination status of pigs included in this study is unknown. However, Ingelvac PRRS^®^ MLV and Ingelvac CircoFLEX^®^ are the most commonly reported vaccines used for PRRSV and PCV-2, respectively, with 65% and 76% of farmers reporting using them at some stage on-farm (frequency of use, proportion of animals vaccinated, age of animals at vaccination, and individual vaccine records are not available).

The present cross-sectional study commenced on the 3 February 2020. First, all farmers were contacted individually by a researcher (S.C.C.) via a phone call. Of the 40 active farms, 73% (29/40) agreed to participate in the present study, with data collection visits occurring from 2 March 2020 to 11 March 2021 (see pig samples below).

All animal samples were collected in compliance with the HKSAR guidelines for research on animals, with relevant animal licenses and animal ethics approval: City University Animal Ethics Approval, A-0402 (Improving Pig Health and Production in Hong Kong).

### 2.2. Pig Samples

Sample collection utilized a two-stage cluster sampling method. Firstly, farms were selected based on the farmers’ willingness to participate the study; secondly, stratified random sampling was used within each of the clusters (farms). Each of the farms included in the study (*n* = 29) were visited once, with all samples for that farm collected on that day. On each farm, 35 pigs were blood sampled via jugular or cranial vena cava venipuncture, with five pigs selected from each of the following seven age groups: 4 weeks old, 8 weeks old, 12 weeks old, 16 weeks old, 20 weeks old, 24 weeks old and sows. These age groups were selected as they represent each of the key stages of production: 4 weeks old being representative of piglets prior to weaning; 8 weeks old and 12 weeks old being representative of younger and older weaner pigs, respectively; 16 weeks old being representative of grower pigs; 20 weeks old and 24 weeks old being representative of younger and older finisher pigs, respectively; and sows representing the breeding pigs (Figure 1).

The pigs were not individually identified; i.e., no ear tags or other means of identification were used for pigs on these farms. However, the farmers did record the age group of the pigs within each of the pig pens on the farm. Therefore, to sample, the researchers randomly caught a pig from a pen in the target age group, and this process was repeated until five pigs (without replacement, i.e., the pig previously sampled would not be caught again) were sampled in that age group. Wherever possible, pigs were caught from different pens from those available for the age group. However, the researchers did not record the specific pens the pigs came from. The whole procedure was then applied to every age group.

### 2.3. Blood Sample Processing and Testing

Blood samples were transported to the laboratory (CityU Veterinary Diagnostic Laboratory, City University of Hong Kong) within two hours of collection. Sera were obtained by centrifuging the clotted blood samples at 1500 rpm for 10 min, after which the sera were transferred to a 2 mL tube for storage at −30 °C. Nucleic acid extraction was performed using Tianlong™ nucleic acid extractor as per manufacturer instructions and with internal extraction control RNA for quality control. PCR testing was done for each of PRRSV (European and North American strains) and PCV2 using commercially available real-time PCR test kits (genesig^®^ Advanced Kit (Primerdesign Ltd., Chandler’s Ford, United Kingdom); further information about the test kits is available at [32,33]) and following the provided protocol. Positive and negative controls were included for quality control, as is standard practice. Samples were reported as positive when there was positive amplification with the typical sigmoidal shape within 50-cycles, as referenced by the manufacturer. Out of 1015 pig blood samples, there were missing test results for two pigs, as insufficient blood was taken during sampling. These two pigs were excluded from the subsequent analysis.

### 2.4. Statistical Analysis

In the analysis, it was assumed that the pig farms were selected using simple random sampling, and then within each farm, stratified random sampling was used to sample individual pigs. Due to the finite numbers of pigs and farms in Hong Kong, a design-based approach was used to analyze the data, as it allowed direct inferences to be made for the target populations; namely, the population of farmed pigs in the HKSAR (overall and by age group) and pigs on each individual farm. With the design-based approach, the observed PCR result was regarded as a fixed constant, while the inclusion of a pig in the sample was treated as random variable [34].

Prevalences were estimated for each of PRRSV European strain (PRRSV-1), PRRSV North American strain (PRRSV-2) and PCV2 for: (1) the population of farmed pigs in the HKSAR, (2) each age group of farmed pigs and (3) each individual farm in the HKSAR. For the population of farmed pigs and individual farms, the analyses were based on the approach described by Yang and Laven [34]. The 95% confidence intervals (95%CIs) were calculated using normal approximation based on the estimates and their estimated variances. The analyses were conducted in Python, with the code provided in the supplementary material. To estimate the prevalences within age group, data were analyzed using the ‘survey’ package in R [35], with 95%CIs computed using the incomplete beta function and an effective sample size based on the estimated variance of the prevalence, since some prevalence estimates were close to zero [36].

## 3. Results

The prevalences of PRRSV-1, PRRSV-2 and PCV2 in the farmed pig population in the HKSAR were estimated as 7.6% (95%CI: 4.8–10.3%), 12.2% (95%CI: 9.6–14.7%) and 20.3% (95%CI: 14.3–26.2%), respectively.

**Figure 2:**
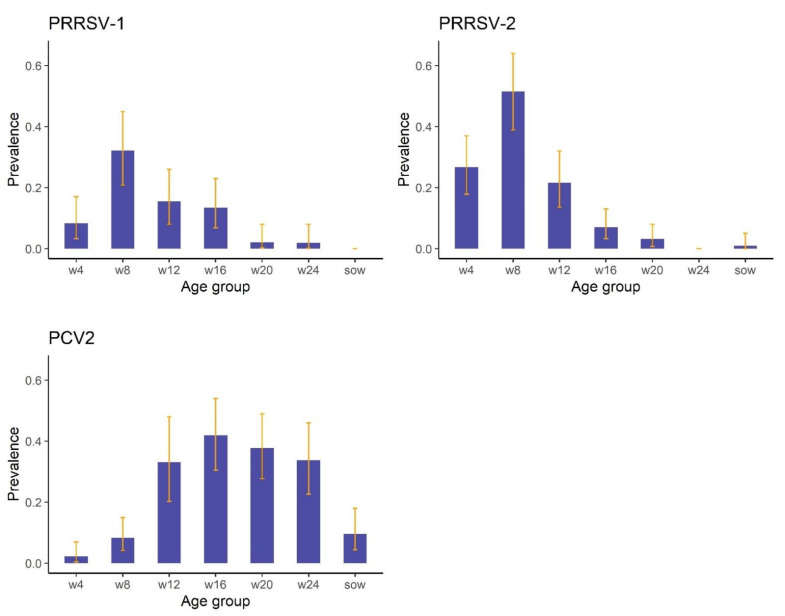
Estimated prevalences including 95%confidence intervals (the error bars) of porcine reproductive and respiratory syndrome virus European strain (PRRSV-1) and North American strain (PRRSV-2) and porcine circovirus type 2 (PCV2) for different age groups of pigs in the HKSAR farmed pig population.w4 = 4-week-old pigs; w8 = 8-week-old pigs; w12 = 12-week-old pigs; w16 = 16-week-old pigs; w20 = 20-week-old pigs; w24 = 24-week-old pigs; sow = breeding sows.

There were differences in estimated prevalence between age groups (Figure 2). For PRRSV-1 and PRRSV-2, there was a trend for increasing prevalence in the younger pigs, with the greatest prevalence estimated for 8-week-old pigs; 32.1% (95%CI: 20.8–45.0%) and 51.5% (95%CI: 38.9–64.0%) for PRRSV-1 and PRRSV-2, respectively (Figure 2). In contrast, increased prevalences of PCV2 were observed in pigs from 12 weeks old to 24 weeks old, with lower prevalences estimated for younger pigs and sows (Figure 2).

Of the 29 farms sampled, 48% (*n* = 14), 86% (*n* = 25) and 79% (*n* = 23) had at least one pig from any age group test positive for PRRSV-1, PRRSV-2 and PCV2, respectively (Table 1). Only two farms had no pigs test positive for both PRRSV-1 and PRRSV-2 strains (Table 1).

**Figure 3:**
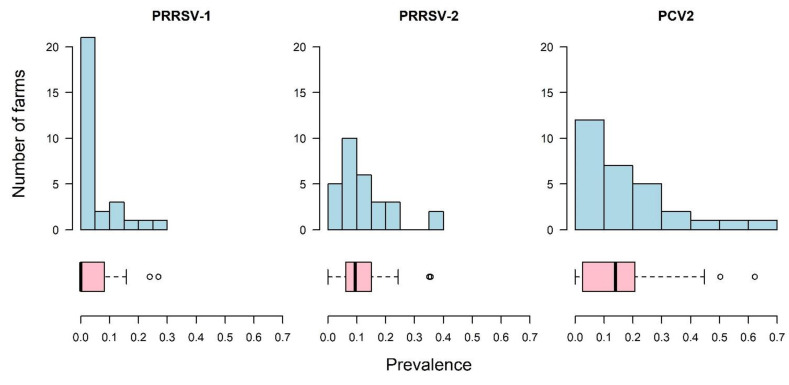
For 29 farms sampled in the HKSAR, the number of farms with, and corresponding prevalence of, porcine reproductive and respiratory syndrome virus European strain (PRRSV-1) and North American strain (PRRSV-2) and porcine circovirus type 2 (PCV2), shown in 10% increments from 0% to 70%.

The within-farm prevalences are shown in Figure 3. With respect to PRRSV-1, only 21% (*n* = 6) of farms had a prevalence ≥10%, compared to PRRSV-2 where 48% (*n* = 14) of farms had a prevalence of ≥10% and PCV2 where 59% (*n* = 17) of farms had a prevalence of ≥10% (Figure 3).

## 4. Discussion

A previous study in the HKSAR reported there had been prior clinical outbreaks of PRRSV, and it was known that both PRRSV-2 and PRRSV-1 strains were present locally [29]. However, the focus of that study was genomic sequencing, and only 16 historical samples (collected during 2003 to 2004) were included [29]. To the authors’ knowledge, ours is the first study to describe the prevalence of PRRSV and PCV2 in the farmed pig population in the HKSAR. We were able to recruit a large proportion of farms to participate in the study (73%). Overall, 48%, 86% and 79% of farms were positive for PRRSV-1, PRRSV-2 and PCV2, respectively, highlighting the need for implementation of robust, individual farm monitoring and preventive health management plans to reduce the likely economic impacts of these pathogens in the HKSAR context.

The prevalences of PRRSV-1 and PRRSV-2 in the farmed pig population in the HKSAR were estimated as 7.5% and 12.2%, respectively. This study included 5 pigs from 7 different age groups, resulting in 35 pigs sampled from each of the 29 farms. This allowed us to examine differences in pathogen prevalence between age groups. Additionally, as one of our target populations for prevalence estimation was the entire farmed pig population in the HKSAR, it was necessary to sample a range of ages representative of different ages and production stages, rather than focusing on pigs within a single age group since prevalence has been reported to vary based on age [11,14]. Similarly, as we were interested in population prevalence, pigs were randomly sampled from within each of these age groups, rather than being selected based on clinical signs or production parameters or rather than utilizing samples that had been collected as part of on-farm diagnostic investigations. As has been previously reported [11], we also found decreased PRRSV prevalence in the older pigs. Interestingly, we also estimated appreciably lower prevalences of PRRSV in 4-week-old pigs compared to 8-week-old pigs. Following weaning (i.e., after the 4-week-old sampling) pigs are shifted from the farrowing house to the weaner shed, so this increase in 8-week-old pigs may be explained by the mixing of infected and non-infected pigs as they were grouped together in the weaner shed. Overall, the high number of farms with at least one positive pig indicates that the majority of farms in the HKSAR would be classified as PRRSV-unstable [37].

The prevalence of PCV2 in the farmed pig population in the HKSAR was 20.3%, similar to that reported in Korea [26] and the USA [21], but lower than that reported in Mainland China where overall prevalence was estimated at 46% [25]. The prevalences of 33.2 to 42.0% estimated for 12-week-old to 24-week-old pigs are similar to those reported by previous studies for comparable age groups of pigs in countries such as Mainland China [25], Thailand [38] and Korea [39]. The prevalence in the sows (9.7%) is lower than the 35% reported by [25]. However, as with the PRRSV comparisons, the methodologies used in the PCV2 prevalence studies vary. For example, many previous studies used biased sampling, utilizing previously collected samples; targeted specific age groups; or selected pigs to sample based on clinical signs [20,21,25,40,41].

As a cross-sectional study, we aimed to understand the current prevalences of these pathogens in the HKSAR. This information can be used to prioritize future pathogen-specific intervention research to minimize their negative economic impacts. This will involve animal- and farm-level risk factor analyses and creation of simulation models investigating their economic consequences. Based on overseas data [5,12,13], the economic consequences of our estimated prevalences, combined with the PRRSV-unstable status, mean these are likely to be significant. Farmers may not be aware of these losses, as the signs associated with these viral infections are non-specific, particularly in an endemic setting [42,43]. This requires further investigation.

One of the key limitations of our cross-sectional study is that farms were not selected using a probability sampling approach. This may bias the prevalence estimates, as farms with weaker biosecurity and management, and hence higher prevalences, may have been more likely to refuse to participate in our survey. Therefore, our survey estimates may underestimate the prevalences. Additionally, the limited vaccine record keeping on farms meant robust data were not available regarding the vaccination status of each of these farms, nor was it known if any of the pigs sampled had been vaccinated, and if so, which vaccine protocol was followed. Thus, vaccination has not been accounted for in the analysis and may have influenced the results. In this study, we used PCR rather than ELISA, enabling us to detect the virus, rather than detecting prior exposure. However, it is important to note that detecting each of these pathogens does not indicate that the animals were diseased, nor were clinical signs evaluated in these pigs.

In this study, very few farms had all pigs test negative. Therefore, it is likely that both PRRSV and PCV2 are industry-wide problems, and objective assessment of on-farm preventive measures is urgently needed. The results of this study can be used to encourage farmers to evaluate the effectiveness of their management strategies for control and prevention of PRRSV and PCV2. These results suggest the current protocols are ineffective, which likely results in substantial production and economic loss in the HKSAR. Useful next steps would involve genetic sequencing of these pathogens to evaluate relatedness and spread, identification of risk factors for infection, and evaluation of individual animal-, farm- and industry-level productivity losses in the HKSAR.

## Figures and Tables

**Table 1:** For 29 farms sampled in the HKSAR, of the 35 pigs sampled on each farm, the actual number of pigs that tested PCR positive for porcine reproductive and respiratory syndrome virus European strain (PRRSV-1) and North American strain (PRRSV-2) and porcine circovirus type 2 (PCV2) and the estimated prevalence (%) of each pathogen within each farm.

	PRRSV-1	PRRSV-2	PCV2
1	3 (3.9%)	4 (5.2%)	3 (7.8%)
2	12 (24.0%)	6 (12.7%)	0 (0.0%)
3	2 (3.5%)	12 (35.6%)	12(14.0%)
4	9 (15.9%)	3 (7.6%)	10 (24.1%)
5	0 (0.0%)	3 (6.2%)	18 (39.9%)
6	0 (0.0%)	8 (9.5%)	1 (1.2%)
7	0 (0.0%)	0 (0.0%)	4 (14.1%)
8	5 (12.4%)	0 (0.0%)	18 (44.9%)
9	13 (26.9%)	3 (6.8%)	0 (0.0%)
10	0 (0.0%)	0 (0.0%)	0 (0.0%)
11	0 (0.0%)	12 (35.1%)	8 (20.8%)
12	0 (0.0%)	9 (12.5%)	9 (20.4%)
13	0 (0.0%)	11 (18.8%)	4 (14.1%)
14	7 (13.1%)	0 (0.0%)	24 (62.3%)
15	0 (0.0%)	5 (8.7%)	14 (28.1%)
16	0 (0.0%)	4 (7.1%)	4 (10.2%)
17	5 (9.5%)	1 (1.9%)	22 (50.3%)
18	0 (0.0%)	8 (14.2%)	10 (11.0%)
19	1 (2.1%)	9 (20.9%)	8 (20.5%)
20	2 (1.8%)	9 (24.4%)	15 (19.0%)
21	0 (0.0%)	8 (13.5%)	5 (7.4%)
22	0 (0.0%)	6 (11.1%)	0 (0.0%)
23	6 (11.8%)	8 (17.7%)	4 (8.5%)
24	4 (8.2%)	6 (12.3%)	8 (16.7%)
25	0 (0.0%)	7 (15.0%)	1 (8.2%)
26	2 (3.6%)	5 (8.9%)	0 (0.0%)
27	2 (4.1%)	4 (7.3%)	1 (2.5%)
28	0 (0.0%)	10 (21.1%)	0 (0.0%)
29	0 (0.0%)	4 (6.1%)	21 (39.0%)

## Data Availability

Inquiries of the original research data can be directed to the corresponding author.

## Supplementary Materials

The following supporting information can be downloaded at: https://www.mdpi.com/article/10.3390/vetsci9020080/s1, Data Statistics.
