# Supplementary material for: First Study to Describe the Prevalence of Porcine Reproductive and Respiratory Syndrome Virus and Porcine Circovirus Type 2 among the Farmed Pig Population in the Hong Kong Special Administrative Region
Source: Vet Sci. 2022 Feb 14;9(2):80. doi: 10.3390/vetsci9020080 (PMC8874621; doi:10.3390/vetsci9020080)
Supplement: Supplementary file 1 [file vetsci-09-00080-s001.zip › vetsci-1574926-supplementary.pdf]

```
# Define functions
import pandas as pd
import numpy as np
from scipy.stats import norm

def psu_strata_prev(df,dis_name,psu,strata):
    pij_dict = {}

    for i in range(1,len(df[psu].unique())+1):
        for j in range(1,len(df[strata].unique())+1):
            t_ij = sum(df[dis_name] [ (df[psu]==i) & (df[strata] == j)])
            p_ij = t_ij / len(df[dis_name] [ (df[psu]==i) & (df[strata] == j)])
            pij_dict.setdefault(i, []).append(p_ij)

    return pij_dict

def psu_strata_total(df,dic):
    tij_dict = {}

    for i in range(len(df)):
        for j in range(len(df.columns)):
            tij_dict.setdefault(i+1,[]).append(df.iloc[i,j] * dic[i+1][j])

    return tij_dict

def psu_total(dic):
    t_i = []

    for key,value in dic.items():
        t_i.append(sum(value))

    return t_i

def psu_prev(df_sample,df_pop,dis_name,psu,strata):
```

```python
    n = len(df_sample[psu].unique())
    sub_df_pop = df_pop.iloc[:n,]
    p_ij = psu_strata_prev(df_sample,dis_name,psu,strata)
    t_ij = psu_strata_total(sub_df_pop,p_ij)
    t_i = psu_total(t_ij)
    m_i = np.sum(sub_df_pop, axis = 1)

    return t_i/m_i

def region_total(a_list,df_pop,n_psu):
    t = sum(a_list) * len(df_pop) / n_psu
    return t

def region_prev(df,val):
    m_sum = 0
    for i in range(len(df)):
        for j in range(len(df.columns)):
            m_sum += df.iloc[i,j]

    p = val / m_sum

    return p

def overall_prev(df_sample,df_pop,dis_name,psu,strata):
    n = len(df_sample[psu].unique())
    sub_df_pop = df_pop.iloc[:n,]
    p_ij = psu_strata_prev(df_sample,dis_name,psu,strata)
    t_ij = psu_strata_total(sub_df_pop,p_ij)
    t_i = psu_total(t_ij)
    t = region_total(t_i,df_pop,n)
    p = region_prev(df_pop,t)
    return p
```

```
def between_var(df_sample,df_pop,dis_name,psu,strata):
    n = len(df_sample[psu].unique())
    N = len(df_pop)
    sub_df_pop = df_pop.iloc[:n,]
    p_ij = psu_strata_prev(df_sample,dis_name,psu,strata)
    t_ij = psu_strata_total(sub_df_pop,p_ij)
    t_i = psu_total(t_ij)
    t = region_total(t_i,df_pop,n)
    var_btw = sum((t_i - t / N)**2) * ((N**2) / n) * (1 - n/ N) * (1 / (n - 1))
    return var_btw

def within_var(df_sample, df_pop, dis_name, psu, strata):

    n = len(df_sample[psu].unique())
    N = len(df_pop)
    sub_df_pop = df_pop.iloc[:n,]
    M_ij_array = np.array(sub_df_pop)

    p_ij = psu_strata_prev(df_sample,dis_name,psu,strata)
    p_ij_array = np.array(list(p_ij.values()))

    m_ij_dict = {}
    for i in range(1,len(df_sample[psu].unique())+1):
        for j in range(1,len(df_sample[strata].unique())+1):
            m_ij = len(df_sample[dis_name] [ (df_sample[psu]==i) & (df_sample[strata] == j)])
            m_ij_dict.setdefault(i, []).append(m_ij)
    m_ij_array = np.array(list(m_ij_dict.values()))

    array_cal = (1 - m_ij_array / M_ij_array) * ((M_ij_array)**2) * ((p_ij_array * (1 -
p_ij_array)) / (m_ij_array - 1))

    var_wt = np.sum(np.sum(array_cal, axis=1)) * N / n

    return var_wt
```

```
def var_prev(df_sample, df_pop, dis_name, psu, strata):
    var_w = within_var(df_sample, df_pop, dis_name, psu, strata)
    var_b = between_var(df_sample,df_pop,dis_name,psu,strata)
    var_t = var_b + var_w
    M = np.sum(np.array(df_pop))
    var_p = var_t / (M**2)
    return var_p

def CI_norm_approx(df_sample, df_pop, dis_name, psu, strata,conf_lv):
    var = var_prev(df_sample, df_pop, dis_name, psu, strata)
    mean = overall_prev(df_sample,df_pop,dis_name,psu,strata)
    SE = np.sqrt(var)
    alpha = 1 - conf_lv
    upper = mean + norm.ppf((1 - alpha / 2))*SE
    lower = mean - norm.ppf((1 - alpha / 2))*SE
    return lower, upper

#Data analysis
df = pd.read_csv("css.csv").dropna()
m = pd.read_csv('M.csv',header=None)
m1 = m.iloc[0:29,]
overall_prev(df,m,'PCV2','farm','age')
CI_norm_approx(df,m,'PCV2','farm','age',0.95)
overall_prev(df,m,'PRRSEU','farm','age')
CI_norm_approx(df,m,'PRRSEU','farm','age',0.95)
overall_prev(df,m,'PRRSUS','farm','age')
CI_norm_approx(df,m,'PRRSUS','farm','age',0.95)

df2 = pd.DataFrame()

for i in range(3):
    df2["v"+str(i+1)] = psu_prev(df,m,df.columns[i],'farm','age')
df2.columns = ['PCV','PRRSEU','PRRSUS']
```

```
df2['PCV2'] = df2['PCV']*1
df3=df2.drop(columns=['PCV'])
df3.to_csv('p_i.csv',index=False)
```
